# Fourth Human Parechovirus Serotype

**DOI:** 10.3201/eid1210.051647

**Published:** 2006-10

**Authors:** Kimberley S.M. Benschop, Janke Schinkel, Manon E. Luken, Peter J.M. van den Broek, Matthias F.C. Beersma, Negassi Menelik, Hetty W.M. van Eijk, Hans L. Zaaijer, Christina M.J.E. VandenBroucke-Grauls, Marcel G.H.M. Beld, Katja C. Wolthers

**Affiliations:** * Academic Medical Center, Amsterdam, the Netherlands;; † Primagen, Amsterdam, the Netherlands;; ‡ Leiden University Medical Center, Leiden, the Netherlands;; § BovenIJ Ziekenhuis, Amsterdam, the Netherlands

**Keywords:** New human parechovirus genotype, HPeV serotype, neutralization assay, full length genotyping, phylogenetic analysis, SimPlot analysis, dispatch

## Abstract

We identified a novel human parechovirus (HPeV) type (K251176-02) from a neonate with fever. Analysis of the complete genome showed K251176-02 to be a new HPeV genotype. Since K251176-02 could not be neutralized with antibodies against known HPeV serotypes 1–3, it should be classified as a fourth HPeV serotype.

Infections with human parechoviruses (HPeVs) are commonly associated with mild gastrointestinal and respiratory symptoms in young children (*1**–**3*), but more severe conditions, such as flaccid paralysis (*4*) and encephalitis (*5*), have also been described. Recently, a new serotype (HPeV3) has been isolated, which has been associated with transient paralysis (*6*) and neonatal sepsis (*7*).

HPeV1 and HPeV2 were previously known as the enteroviruses echovirus 22 and 23 but were reclassified into a new genus within the family *Picornaviridae* after phylogenetic analysis showed that parechoviruses were distinct from other picornaviruses (*1**–**3**,**8**–**11*). HPeVs have predominantly been isolated from young children, and increasing evidence shows that HPeV can cause serious illness in these patients.

We recently showed that infection with HPeV3 is associated with younger age and more severe disease than is infection with HPeV1 (*12*). During the screening of patient samples, we identified 1 aberrant HPeV type. Phylogenetic analysis of the full-length sequence and viral neutralization assays showed that the isolate designated K251176-02 is a new HPeV genotype and serotype.

## The Study

Viral culture of the stool of a 6-day-old patient with a 2-day history of high fever and poor feeding and no history of gastrointestinal or respiratory symptoms showed enterovirus cytopathic effects. However, PCR targeted at the 5´ untranslated region (UTR) of enterovirus (*13*) was negative, whereas a 5´ UTR PCR specific for HPeV (*12*) was positive.

Results of sequencing the VP1 region (*12*) suggested that K251176-02 was a novel HPeV genotype. Therefore, the full-length sequence was determined. Combinations of consensus primers were used to generate partially overlapping amplicons that covered the complete genome. Amplicons were sequenced according to a primer walking strategy. The 5´ UTR was amplified by using the 5´ RACE System (Invitrogen, Carlsbad, CA, USA). Because a primer composed of the first 22 nucleotides (nt) of published consensus parechovirus sequences was used to amplify the 5´ UTR proximal end, these 22 nt could not be determined with absolute certainty (*8*). The 3´ UTR end was amplified with a tagged oligo-dT primer.

The complete genome of K251176-02 was 7,348 nt long, containing a 5´ UTR of 708 nt, a large single open reading frame (ORF) of 6,549 nt, and a 3´ UTR of 91 nt followed by a poly(A) tract. The full-length sequence of K251176-02 has been deposited in GenBank under accession no. DQ315670.

**Figure 1:**
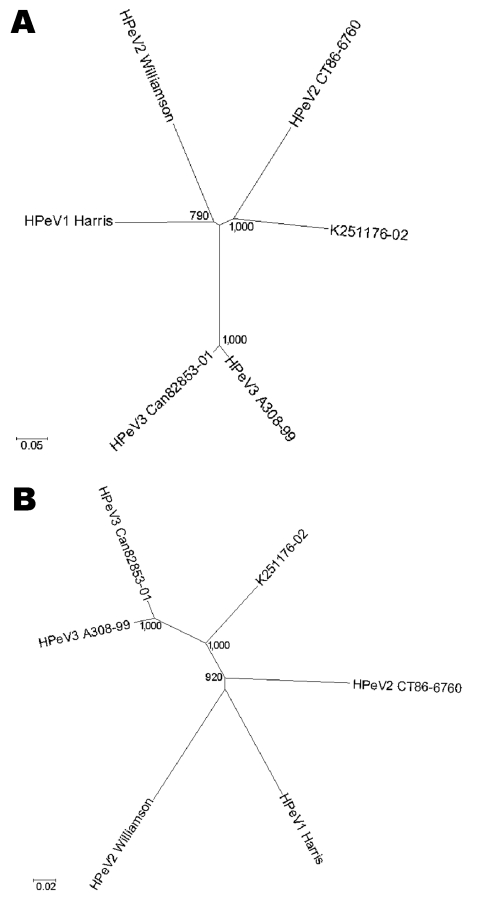
Unrooted phylogenetic trees showing the relationship between K251176-02 (DQ315670) and the prototype strains human parechovirus serotype 1 (HPeV1) Harris (S45208), HPeV2 Williamson (AJ005695), HPeV2 CT86-6760 (AF055846), HPeV3 A308-99 (AB084913), and Can82853-01 (AJ889918) based on nucleotide Jukes and Cantor substitution model for the capsid region (A) and the nonstructural region (B). The tree was constructed by the neighbor-joining method as implemented in MEGA version 3.1. Gaps introduced for optimal alignment were not considered informative and were excluded from the analyses by complete deletion. Numbers represent the frequency of occurrence of nodes in 1,000 bootstrap replicas. The use of other evolution models did not influence the tree topology.

We found a best-match nucleotide identity (*14*) of 72.2% in the VP1 gene with HPeV2 CT86-6760, which suggests that K251176-02 is most closely related to HPeV2 CT86-6760. Indeed, phylogenetic analysis of the capsid nucleotide sequence based on Jukes and Cantor distances showed K251176-02 to cluster with HPeV2 CT86-6760 (Figure 1A). However, the genetic distance was considerable (0.327) and comparable to the genetic distance between HPeV1 Harris and HPeV2 Williamson (0.332). Phylogenetic analysis of the nonstructural region showed that K251176-02 clustered with the HPeV3 prototypes A308-99 and Can82853-01 (Figure 1B).

**Figure 2:**
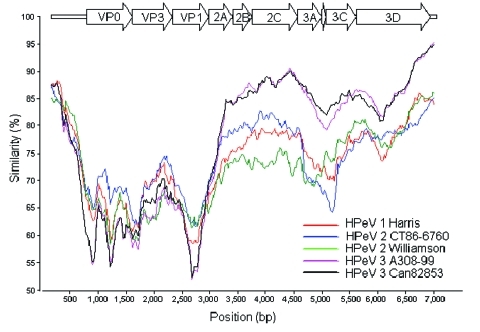
Similarity plot of human parechovirus serotype 1 (HPeV1) Harris (S45208), HPeV2 Williamson (AJ005695), HPeV2 CT86-6760 (AF055846), HPeV3 A308-99 (AB084913), and Can82853-01 (AJ889918) against K251176-02. Each curve is a comparison between the K251176-02 genome and an HPeV prototype. Each point represents the percentage identity within a sliding window 600 bp wide, centered on the position plotted, with a step size of 20 bp. Positions containing gaps were excluded from the comparison by gap stripping, and Jukes and Cantor correction was applied. Similarity plots of the full-length sequences of the HPeV prototypes were generated by using SimPlot version 2.5.

To identify recombination events between the different HPeV prototypes, a SimPlot analysis was performed on the known full-length nucleotide HPeV genomes against K251176-02. The SimPlot analysis (Figure 2) showed the differential similarity of K251176-02 with HPeV2 CT86-6760 in the highly variable P1 region and with HPeV3 in the more conserved P2–P3 region. This finding may be the result of a recombination event.

The secondary structure of the 5´ UTR of K251176-02, determined by the *Mfold* program of Zuker and Turner (http://mfold2.wustl.edu), was predicted to be highly structured and was characterized by a stable hairpin at the proximal end that was also found in known HPeV prototypes (8, 11, data not shown). The predicted secondary structure of the 3´ UTR of K251176-02 contained the same 1-stem loop organization as the HPeV prototypes and was similar to the secondary structure of HPeV1 Harris and HPeV2 Williamson and CT86-6760 (*15*).

A comparison of the complete ORF of K251176-02 with the HPeV prototypes showed an amino acid identity of 86.9% to 90.1% (Table 1). This amount is in the same range of amino acid identity as observed between known HPeV protoypes. For the VP1 gene, the greatest amino acid identity was observed with HPeV2 CT86-6760 (80.4%). In the nonstructural region, identity was greater to HPeV3, with 98.1% identity in the polymerase gene (3D^pol^).

**Table 1:** Amino acid identity matrix of all known human parechovirus (HPeV) full-length sequences*

Target		HPeV1 (H)	HPeV2 (W)	HPeV2 (CT)	HPeV3 (A308)	HPeV3 (Can)
ORF						
	K251176-02	88.7	86.9	90.1	89.1	89.3
	HPeV1 (H)	–	88.6	88.1	87.2	87.3
	HPeV2 (W)	–	–	85.8	85.1	85.0
	HPeV2 (CT)	–	–	–	86.7	86.9
	HPeV3 (A308)	–	–	–	–	98.2
	HPeV3 (Can)	–	–	–	–	–
P1						
	K251176-02	78.3	78.6	81.3	74.7	75.1
	HPeV1 (H)	–	81.6	76.4	74.9	75.3
	HPeV2 (W)	–	–	74.2	73.9	73.9
	HPeV2 (CT)	–	–	–	73.0	73.5
	HPeV3 (A308)	–	–	–	–	97.4
	HPeV3 (Can)	–	–	–	–	–
P2-P3						
	K251176-02	94.3	91.5	94.9	97.0	97.1
	HPeV1 (H)	–	92.4	94.5	93.8	93.9
	HPeV2 (W)	–	–	92.2	91.2	91.1
	HPeV2 (CT)	–	–	–	94.2	94.2
	HPeV3 (A308)	–	–	–	–	98.6
	HPeV3 (Can)	–	–	–	–	–

*Amino acid identities for the open reading frame (ORF), capsid region (P1), and nonstructural region (P2-P3) are based on p-distance models between K251176-02 (DQ315670) and the HPeV prototypes, HPeV1 (H) (Harris, S45208), HPeV2 (W) (Williamson, AJ005695), HPeV2 (CT) (CT86-6760, AF055846), HPeV3 (A308) (A308-99, AB084913), and HPeV3 (Can) (Can82853-01, AJ889918). The full-length sequence of K251176-02 was aligned with the HPeV prototypes by using ClustalW, included in the Vector NTI Advance 10 software package (Invitrogen, Carlsbad, CA, USA). Alignment was edited by using GeneDoc software (version 2.6.02). The matrix was constructed by using MEGA version 3.1. The 5´ untranslated region (UTR) and 3´ UTR are excluded from the analysis because the regions are noncoding.

Comparison of the deduced amino acid sequence in the capsid region of K251176-02 with the HPeV prototypes showed that the sequences that are predicted to be part of the β-barrel structure (*6**,**10**,**11*) are well conserved in K251176-02. Like HPeV1 and HPeV2, K251176-02 also contained an RGD motif at the C-terminal end of the VP1 gene, which was absent in HPeV3 (*6**,**7**,**15*). K251176-02 also contained the common motifs X_2_GXGK(S/T) and DDLXQ (2C gene), which are predicted to have a helicase function. The active-site cysteine of the protease 3C is in the context of GXCG, and the active site of polymerase 3D^pol^ contains the conserved sequence YGDD. The well-conserved motifs within the 3D^pol^ gene (KDELR, PSG, and FLKR) were also found in K251176-02 (*6**,**9**,**11*).

In summary, K251176-02 represents a new genotype in the genus *Parechovirus*. To confirm that K251176-02 is also a new serotype, a neutralization assay was performed. Table 2 shows that K251176-02 could not be neutralized by antisera directed against HPeV1 Harris, HPeV2 Williamson, and HPeV3 A308-99, which confirms that K251176-02 is a new genotype that can be classified as a fourth HPeV serotype.

**Table 2:** Neutralization assay with LLcMk2 cells*

Virus	Antiserum			Viral controls
	a-HPeV1 (Harris)	a-HPeV2 (Williamson)	a-HPeV3 (A308-99)	
HPeV1 Harris	–	++++	++++	++++
HPeV2 Williamson	++++	–	++++	++++
K251181-02 (HPeV3)	++++	++++	–	++++
K251176-02	++++	++++	++++	++++

*Culture isolates of K251176-02, human parechovirus 1 (HPeV1, echovirus 22) and HPeV2 (echovirus 23) from a reference panel (National Institute for Public Health and the Environment, Bilthoven, the Netherlands) and K251181-02 that was previously genotyped as HPeV3 (*12*) were incubated with antisera (20 U/mL in Eagle minimal essential medium) directed against HPeV1 Harris, HPeV2 Williamson, and HPeV3 A308-99. The antisera to HPeV1 and HPeV2 were raised in horses. The antiserum to HPeV3 was raised in guinea pigs. Neutralization is done on a 96-microtiter plate containing a monolayer of LLcMk2 cells that have been incubated for 3 days. The assay was determined after viral controls (no antisera used) of the 4 culture isolates showed cytopathic effects >50% (++++).

## Conclusions

HPeVs are classified in the genus *Parechovirus* in the family *Picornaviridae*. The recently identified HPeV3 has been associated with severe illness in young children in several studies (*6**,**7**,**12*). This association has increased the awareness of HPeVs as relevant pathogens in young children.

We identified a new HPeV genotype in a stool specimen from a neonate with high fever. Since classification criteria based on genotyping have not been defined for HPeVs, we used the criteria proposed by Oberste et al. (*14*) for the classification of new enteroviral genotypes. According to these criteria, a new genotype is defined when a best-match nucleotide identity of <70% is found in the VP1 gene. A 70%–75% best-match nucleotide identity indicates further characterization is needed. Therefore, neutralization assays were conducted; these assays showed that K251176-02 did not neutralize with antisera directed against the 3 known HPeV serotypes. This finding indicates that K251176-02 is a new genotype that can be classified as a fourth HPeV serotype.

The patient from whom K251176-02 was isolated had high fever but no signs of neonatal sepsis, as has been found in infections with HPeV3 (*6**,**7**,**12*). Previous data suggest differences in severity of disease between the different HPeV serotypes (*12*); however, more data are needed to elucidate epidemiologic and pathogenic features of the different HPeV serotypes, including K251176-02.

HPeV2 CT86–6760 was genotypically as distinct from HPeV2 Williamson as from other HPeV types (Table 1). The existence of 2 genotypically divergent HPeV serotypes 2 is surprising and needs to be elucidated further. This finding, however, argues in favor of a universal typing method that is based on molecular characteristics (genotyping) instead of serotyping, provided classification criteria are well defined.
